# The Fence or the Ambulance?

**Published:** 1912-06

**Authors:** Joseph Malins


### The Fence or the Ambulance?

(BY JOSEPH MALINS.)

'Twas a dangerous cliff, as they freely confessed.
Though to walk near its crest was so pleasant;
But over its terrible edge there had slipped
A duke and full many a peasant.
So the people said something would have to be done,
But their projects did not at all tally.
Some, "Put a fence around the edge of the cliff."
Some, "An ambulance down in the valley."

But the cry for the ambulance carried the day,
And it spread through the neighboring city;
A fence may be useful or not, it is true,
But each heart became brimful of pity
For those who slipped over that dangerous cliff.
And the dwellers in highway and alley
Gave pounds or gave pence, not to put up a fence,
But an ambulance down in the valley.

Then an old sage remarked: "It's a marvel to me
That people give far more attention
To repairing results than to stopping the cause,
When they'd better aim at prevention.
Let us stop at its source all this mischief," cried he.
"Come, neighbors and friends, let us rally;
If the cliff we will fence, we might almost dispense
With the ambulance down in the valley."

"Oh, he's a fanatic," the other rejoined;
"Dispense with the ambulance? Never!
He'd dispense with all charities, too; if he could
No, no, we'll support them forever!

